# Cost-Reference Particle Filter-Based Method for Constructing Effective Brain Networks: Application in Optically Pumped Magnetometer Magnetoencephalography

**DOI:** 10.3390/bioengineering11121258

**Published:** 2024-12-12

**Authors:** Yuyu Ma, Xiaoyu Liang, Huanqi Wu, Hao Lu, Yong Li, Changzeng Liu, Yang Gao, Min Xiang, Dexin Yu, Xiaolin Ning

**Affiliations:** 1 Key Laboratory of Ultra-Weak Magnetic Field Measurement Technology, Ministry of Education, School of Instrumentation and Optoelectronic Engineering, Beihang University, 37 Xueyuan Rd., Haidian District, Beijing 100083, China; ma_yuyu@buaa.edu.cn (Y.M.); whqmeg@buaa.edu.cn (H.W.); luhao66@buaa.edu.cn (H.L.); by2017335@buaa.edu.cn (Y.L.); by2143115@buaa.edu.cn (C.L.); yanggao@buaa.edu.cn (Y.G.); xiang_min@buaa.edu.cn (M.X.); 2 Hangzhou Institute of National Extremely-Weak Magnetic Field Infrastructure, 465 Binan Rd., Binjiang District, Hangzhou 310051, China; 3 Hefei National Laboratory, 96 Jinzhai Rd., Gaoxin District, Hefei 230088, China; 4 Shandong Key Laboratory for Magnetic Field-Free Medicine & Functional Imaging, Institute of Magnetic Field-Free Medicine & Functional Imaging, Shandong University, 27 South Shanda Rd., Licheng District, Jinan 250100, China; yudexin0330@163.com

**Keywords:** OPM-MEG, effective brain network, Granger causality, cost-reference particle filter

## Abstract

Optically pumped magnetometer magnetoencephalography (OPM-MEG) represents a novel method for recording neural signals in the brain, offering the potential to measure critical neuroimaging characteristics such as effective brain networks. Effective brain networks describe the causal relationships and information flow between brain regions. In constructing effective brain networks using Granger causality, the noise in the multivariate autoregressive model (MVAR) is typically assumed to follow a Gaussian distribution. However, in experimental measurements, the statistical characteristics of noise are difficult to ascertain. In this paper, a Granger causality method based on a cost-reference particle filter (CRPF) is proposed for constructing effective brain networks under unknown noise conditions. Simulation results show that the average estimation errors of the MVAR model coefficients using the CRPF method are reduced by 53.4% and 82.4% compared to the Kalman filter (KF) and maximum correntropy filter (MCF) under Gaussian noise, respectively. The CRPF method reduces the average estimation errors by 88.1% and 85.8% compared to the MCF under alpha-stable distribution noise and the KF method under pink noise conditions, respectively. In an experiment, the CRPF method recoversthe latent characteristics of effective connectivity of benchmark somatosensory stimulation data in rats, human finger movement, and auditory oddball paradigms measured using OPM-MEG, which is in excellent agreement with known physiology. The simulation and experimental results demonstrate the effectiveness of the proposed algorithm and OPM-MEG for measuring effective brain networks.

## 1. Introduction

Magnetoencephalography (MEG) records magnetic field signals generated by neural electrical activity in the brain. Commercially available MEG systems based on superconducting quantum interference devices (SQUID-MEG) need to operate at acryogenic temperature, making the equipment bulky and expensive to maintain. Recently, advancements in quantum technology have led to the development of novel MEG systems based on optically pumped magnetometers (OPM-MEG) [1,2,3]. OPM-MEG systems operate at room temperature, offering a lightweight, flexible, and wearable solution. Their cost-effectiveness and versatility make them suitable for a broader range of settings and patient populations. OPM-MEG holds significant potential for measuring crucial neuroimaging characteristics such as effective brain networks [4].

Effective networks describe the cause–effect relationships and information flow between brain regions, providing insights that closely align with the brain’s actual information processing mechanisms [5,6]. Granger causality (GC) is a widely used method for constructing effective brain networks, based on the multivariate autoregressive (MVAR) model for time series analysis [7,8]. The coefficients of the MVAR model are typically estimated using the least squares method, which requires data stationarity as a necessary condition for accurate estimation. However, brain neural signals are dynamically non-stationary and exhibit significant variability over time [9]. To address the challenges of applying MVAR to dynamic time–frequency brain networks, a common approach is to use the sliding time window method, under the assumption that the signals are stationary within short time windows [10]. Nevertheless, there is no definitive solution regarding the optimal length of the window, and whether the length matches the underlying neurophysiological processes remains debatable [11]. Furthermore, the least squares method is notably sensitive to noise. For signals with low signal-to-noise ratios, it tends to amplify the effects of noise, which leads to considerable estimation errors in the MVAR model coefficients, resulting in spurious connections during brain network construction [12].

Recently, the Kalman filter has been applied to estimate the coefficients of MVAR models, resulting in effective brain networks with superior dynamic performance compared to those constructed using the least squares method [13,14]. However, the Kalman filter provides optimal linear unbiased estimates under the assumption of Gaussian noise. Actual neural signals frequently contain non-Gaussian noise, which compromises the optimality of the Kalman filter’s estimates and may reduce estimation accuracy [15]. Additionally, the statistical characteristics of the noise are generally unknown, which poses difficulties for the Kalman filter in estimating MVAR model coefficients.

To address the issue of poor coefficient estimation accuracy in the MVAR model under unknown noise characteristics, the Granger causality method based on the cost-reference particle filter (CRPF-GC) is proposed. The estimation performance of CRPF-GC is evaluated through simulations under Gaussian noise, non-Gaussian alpha-stable distribution noise, and pink noise conditions. The results are compared with the Granger causality methods based on the Kalman filter and maximum correntropy filter. In this study, The benchmark data for the effectiveness and reliability assessment of the CRPF-GC method are provided via electroencephalogram (EEG) of somatosensory stimulation in rats. On the other hand, the OPM-MEG system was used to measure finger movement and auditory oddball experiments in humans, and the CRPF-GC algorithm was then applied to construct effective brain networks.

## 2. Materials and Methods

### 2.1. CRPF-GC Method

#### 2.1.1. MVAR Model for Granger Causality Method

The Granger causality method uses the MVAR model to calculate the causal relationships between multidimensional time series. Suppose at time *t*, the *N*-dimensional time series is represented as follows:

(1) $$X_{t}={\left[x_{1,t},x_{2,t},\ldots ,x_{N,t}\right]}^{T}$$

where *T* represents the transpose of variables. The p-order MVAR model for the time series $X_{t}$ can be expressed as follows:

(2) $$\left(\begin{array}{c} x_{1,t}\\ x_{2,t}\\ \vdots \\ x_{N,t} \end{array}\right)=A_{1}\left(\begin{array}{c} x_{1,t-1}\\ x_{2,t-1}\\ \vdots \\ x_{N,t-1} \end{array}\right)+A_{2}\left(\begin{array}{c} x_{1,t-2}\\ x_{2,t-2}\\ \vdots \\ x_{N,t-2} \end{array}\right)+\cdots +A_{p}\left(\begin{array}{c} x_{1,t-p}\\ x_{2,t-p}\\ \vdots \\ x_{N,t-p} \end{array}\right)+\left(\begin{array}{c} \varepsilon_{1,t}\\ \varepsilon_{2,t}\\ \vdots \\ \varepsilon_{N,t} \end{array}\right)$$

That is:

(3) $$X_{t}=\sum_{r=1}^{p}A_{r,t}X_{t-r}+E_{t}$$

where $E_{t}$ is the noise process, and $A_{r,t}$ is the model parameters of MVAR:

(4) $$A_{r,t}=\left[\begin{array}{cccc} a_{11,t-r} & a_{12,t-r} & \cdots & a_{1N,t-r}\\ \vdots & \vdots & \vdots & \vdots \\ \vdots & \vdots & a_{ij,t-r} & \vdots \\ \vdots & \vdots & \vdots & \vdots \\ a_{N1,t-r} & \cdots & a_{N\left(N-1\right),t-r} & a_{NN,t-r} \end{array}\right]$$

where the elements $a_{ij,t-r}$ in the model coefficients $A_{r,t}$ represent the interaction of the *j*-th dimension time series $x_{j}$ on the *i*-th dimension time series $x_{i}$ at time t−r.

#### 2.1.2. MVAR Model Coefficient Estimation Based on CRPF

In this study, the elements of the model coefficients $A_{r,t}$ in the MVAR model are considered as state variables, while the MEG signals are treated as measurement variables. The following state equation and measurement equation are established:

(5) $$a_{t}=a_{t-1}+w_{t}$$

(6) $$x_{t}=H_{t}a_{t}+v_{t}$$

where $a_{t}$ represents the elements in model coefficient matrix $A_{r,t}$, $x_{t}$ is the MEG signal measured at time *t*, $H_{t}={\left(x_{t-1},\;x_{t-2},\ldots ,x_{t-p}\right)}^{T}$ is the observation matrix, and $w_{t}$ and $v_{t}$ are the state noise and measurement noise, respectively.

In practical applications, the statistical characteristics of noise are challenging to determine. To address this issue, this study introduces the CRPF method for estimating the model coefficients of the MVAR model. The CRPF method guides the particle distribution by incorporating a cost function, enabling estimation without prior knowledge of the statistical characteristics in process noise and measurement noise [16]. The CRPF method has been successfully applied in various domains, including frequency-modulated signal detection [17], radar tracking [18], and attitude estimation [19].

The CRPF method iteratively updates the state variables and variance in the absence of prior information by designing a cost function and a risk function. To facilitate iterative calculations, the cost function with a recursive additive structure is employed. At time *t*, the cost function of the *i*-th particle is designed as follows [16]:

(7) $$C_{t}^{i}=\lambda C_{t-1}^{i}+\Delta C_{t}^{i}$$

(8) $$\Delta C_{t}^{i}=\Delta C\left(a_{t}^{i}|x_{t}\right)={∥x_{t}-H_{t}\cdot a_{t}^{i}∥}_{2}^{q}$$

where λ (0<λ<1) is the forgetting factor, which is used to balance the inheritance and prediction costs. *q* (q≥1) is a normal number. A large *q* value means that the sampling particles are centered on a few of the most important particles. The *i*-th particle risk function at time t is defined as follows:

(9) $$R_{t}^{i}=\lambda C_{t-1}^{i}+{∥x_{t}-H_{t}\cdot a_{t-1}^{i}∥}_{2}^{q}$$

As particles approach the true state value, the estimation error decreases, resulting in a smaller cost function value. Consequently, the probability of selecting and retaining such particles increases, which in turn enhances their weights. The approach allows for more effective retention of high-quality particles and mitigates the particle degradation problem. The CRPF algorithm primarily consists of four steps to achieve state variable estimation of the MVAR model parameters.

(1) Initialization

Assuming an initial number of particles *M*, an initial particle set $\left\{a_{0}^{1},a_{0}^{2},\cdots ,a_{0}^{M}\right\}$ is generated. At the initial time point, it is assumed that each particle has an equal cost, set as $C_{0}^{i}=0$. This results in a sample-cost set ${\left\{\left(a_{0}^{i},C_{0}^{i}\right)\right\}}_{i=1}^{M}$.

(2) Resampling

The risk is computed using Equation (8). Based on the risk associated with all particles at time *t*, a monotonically decreasing probability density function is designed to map the risk and is used as the resampling weight:

(10) $${\hat{\pi }}_{t}^{i}\propto \mu_{1}\left(R_{t}^{i}\right)=\frac{{\left(R_{t}^{i}-\underset{j\in \{1,\ldots ,M\}}{min}R_{t}^{j}+\delta \right)}^{-\beta }}{\sum_{l=1}^{M}{\left(R_{t}^{i}-\underset{j\in \{1,\ldots ,M\}}{min}R_{t}^{j}+\delta \right)}^{-\beta }}$$

Here, δ and β (β>0) are empirical parameters. As δ decreases and β increases, the resampling weights for particles with lower risk become larger. A small value of δ also ensures that the denominator remains non-zero. Similar to traditional particle filtering, the CRPF algorithm implements particle selection and retention through the resampling process. During resampling, particles with lower costs are duplicated, while those with higher costs are discarded. The smaller the risk of a particle, the more important it is for state estimation, leading to a higher probability of being selected during resampling. After resampling, a new particle cost set ${\left\{\left({\overset{ˇ}{a}}_{t}^{i},{\overset{ˇ}{C}}_{t}^{i}\right)\right\}}_{i=1}^{M}$ is formed.

(3) Particle Propagation

For i=1,2,…,M, the states of the particles at time *t* are updated using a Gaussian density function with an adaptively chosen variance:

(11) $$a_{t}^{i}∼N\left(\left(H_{t}\cdot {\overset{ˇ}{a}}_{t}^{i}\right),\sigma_{t}^{2,i}I\right)$$

where $\sigma_{t}^{2,i}$ is the variance, given by:

(12) $$\sigma_{t}^{2,i}=\frac{t-1}{t}\sigma_{t-1}^{2,i}+\frac{{∥a_{t}^{i}-{\overset{˘}{a}}_{t}^{i}∥}^{2}}{t\times dim[a]}$$

where dim[a] denotes the dimensionality of the state variables. *I* is the identity matrix with dimensions matching those of the state variables. In the CRPF algorithm, the variance of the propagation density is adjusted online.

(4) State Estimation

The costs at time *t* are calculated using Equations (7) and (8), followed by the computation of the normalized probability density function:

(13) $$\pi_{t}^{i}\propto \mu_{2}\left(C_{t}^{i}\right)=\frac{{\left(C_{t}^{i}-\underset{j\in \{1,\ldots ,M\}}{min}C_{t}^{j}+\delta \right)}^{-\beta }}{\sum_{l=1}^{M}{\left(C_{t}^{i}-\underset{j\in \{1,\ldots ,M\}}{min}C_{t}^{j}+\delta \right)}^{-\beta }}$$

Then, the estimation of the state variable at time *t* is as follows:

(14) $${\hat{a}}_{t}=\sum_{i=1}^{M}\pi_{t}^{i}a_{t}^{i}$$

The information transfer between brain regions relies on neural oscillations, which introduces frequency domain considerations in effective brain network calculations. This study further employs the squared partial directed coherence (sPDC) to measure the time–frequency connectivity among nodes in the brain network. The sPDC value from time series *j* to time series *i* is defined as follows [20]:

(15) $$sPDC_{ij}\left(f\right)=\frac{{\left|\Lambda_{ij}\left(f\right)\right|}^{2}}{\sum_{m=1}^{N}{\left|\Lambda_{mj}\left(f\right)\right|}^{2}}$$

where $\Lambda_{ij}\left(f\right)=\sum_{r=1}^{p}A_{r,t}z^{-k}$, $z=e^{-i2\pi f}$.

#### 2.1.3. Simulation Experiment

To verify the accuracy and reliability of the KF-GC algorithm, a simulation model with a three-dimensional MVAR process was set up, referring to previous studies [21,22], which is represented as follows:

(16) $$\begin{array}{cc} & s_{1,t}=0.59s_{1,t-1}-0.20s_{1,t-2}+c_{t}s_{2,t-1}+d_{t}s_{3,t-1}+v_{1,t}\\ & s_{2,t}=1.58s_{2,t-1}-0.96s_{2,t-2}+v_{2,t}\\ & s_{3,t}=b_{t}s_{1,t-1}+0.60s_{3,t-1}-0.91s_{3,t-2}+v_{3,t} \end{array}$$

where $b_{t}$, $c_{t}$, and $d_{t}$ simulate three variations of model parameters: changes from 0 to a constant value, oscillating changes, and linear changes, respectively. The causal interaction was simulated over a duration of 50 s, with the sampling frequency set at 1000 Hz and model order p=2.

The Gaussian noise and non-Gaussian alpha-stable noise were used in the observation noise $v_{t}$ in order to evaluate the performance of the CRPF-GC algorithm in estimating the MVAR model coefficients. Under the Gaussian noise condition, $v_{t}$ was set to $v_{t}∼N\left(0,R\right)$. Non-Gaussian noise was simulated using an alpha-stable distribution and pink noise.

An alpha-stable distribution is characterized by its stability in probability distributions and its heavy-tailed probability density function. The characteristic function of the alpha-stable distribution is defined as follows [15,23]:

(17) $$\varphi \left(t\right)=\exp\left\{\begin{array}{c} jat-\gamma |t{|}^{\alpha }\left[1+j\beta \mathrm{sgn}\left(t\right)\omega \left(t,\alpha \right)\right] \end{array}\right\}$$

where $\omega \left(t,\alpha \right)=\left\{\begin{array}{c} \tan(\frac{\alpha \pi }{2}),\alpha \neq 1\\ \frac{2}{\pi }\log|t|,\alpha =1 \end{array}\right.$, sgn (*t*) is the sign function, defined as follows:

(18) $$\mathrm{sgn}\left(t\right)=\left\{\begin{array}{cc} 1, & t>0\\ 0, & t=0\\ -1, & t<0 \end{array}\right.$$

And α, β, γ, and *a* are the four parameters of alpha-stable distribution, set as α=1.00, β=0.00, γ=1.00, and α=0.75. Pink noise is a signal with a 1/f spectrum, where the relationship between the power spectral density S(f) and frequency *f* is given by the following:

(19) $$S\left(f\right)\propto \frac{1}{f}$$

In this study, the pink noise was generated by filtering white noise.

The estimation performance of CRPF-GC was compared with the Granger causality methods based on the Kalman filter (KF) [14] and maximum correntropy filter (MCF) [24]. The MCF method is considered to have good estimation performance under non-Gaussian noise conditions. In both KF and MCF, the covariances of the process noise and observation noise were set to 10 and $10^{4}$, respectively. The initial values of the model coefficient matrix $a_{t}$ and the covariance matrix were set to zero matrices, while the kernel width in MCF was set to 50. In the CRPF, the number of particles was set to 1000 (further discussion about particle numbers is provided in the Supplementary Materials). The initialization value was set to $10^{-4}$, the variance $\sigma_{t}^{2}$ was set to $2.5\times 10^{-2}$, and an initial cost $C_{t}$ was set to zero matrices.

The estimation error is used for the comparison between the estimated values and the ground truth values as follows:

(20) $$\mathrm{error}=\frac{1}{N}\sum_{t=1}^{N}\sqrt{{\left(e_{t}-{\hat{e}}_{t}\right)}^{2}}$$

where $e_{t}$ and ${\hat{e}}_{t}$ represent the ground truth values and estimated values, respectively.

### 2.2. Benchmark Somatosensory Stimulation Data in Rats

To evaluate the validity and reliability of the CRPF-GC method, the benchmark EEG data from unilateral whisker stimulation in rats [25] was used for testing the objective performance in effective brain networks. All animal handling procedures were in accordance with Swiss Federal laws. In the experiments, the rats were anesthetized with isoflurane, and 15-channel electrodes were positioned on the cranium. The EEG signals were recorded at a sampling rate of 2000 Hz with an online band-pass filter applied between 1 and 500 Hz. These data are freely available online (https://osf.io/fd5ru, accessed on 20 September 2024), and further details regarding the recording procedure can be found elsewhere [25,26].

The physiology of unilateral whisker stimulation in rats has been extensively studied. In previous studies, the latency of maximum event-related potential (ERP) is 13.9 ms, and increased connectivity in the gamma frequency band (40–90 Hz) has been observed in the contralateral primary somatosensory cortex (cS1) with 5–20 ms [25,26]. In this study, EEG data from right-side whisker stimulation were selected for analysis. Single-trial EEG data from 10 rats were first downsampled to 1000 Hz. The CRPF-GC method was then employed to calculate MVAR model coefficients for the 15 recording channels, with the model order set to the previously reported optimal value (p=4) [26]. The sPDC values were used to calculate the connectivity.

### 2.3. OPM-MEG Experiment and Data Processing

#### 2.3.1. OPM-MEG System

**Figure 1:**
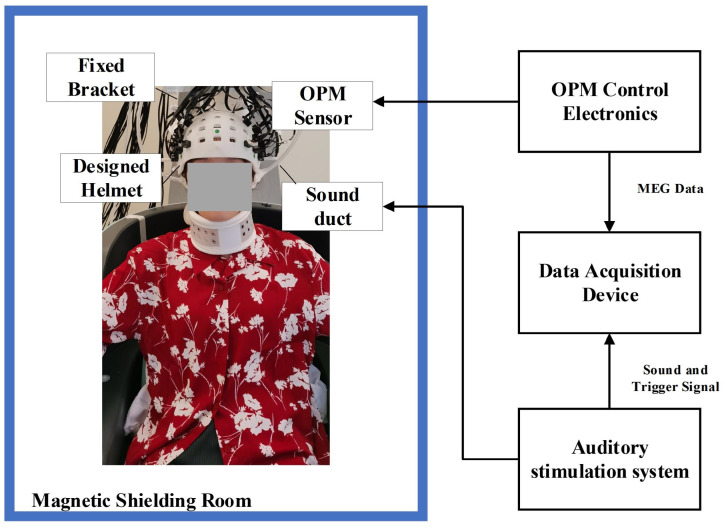
OPM-MEG system.

In this study, a multi-channel MEG system was constructed using OPM magnetometers (QuSpin Inc., Louisville, CO, USA), as illustrated in Figure 1. The OPM magnetometers were placed in the magnetic shielding room (MSR), which maintained a residual magnetic field of less than 10 nT, while the OPM control electronics were located outside the MSR to minimize electromagnetic interference. The auditory stimulation system was controlled by the Psychtoolbox software 3.0.17, and the sound was delivered into the MSR via a plastic duct. The data acquisition device collected MEG signals, auditory signals, and trigger signals, with the sampling frequency set to 1 kHz. In the experiment, the OPM magnetometers were fixed within a rigid helmet to measure the magnetic field components perpendicular to the scalp surface.

#### 2.3.2. Finger Movement Experiments

One subject participated in the finger movement experiment, which was approved by the Research Ethics Committee of Beihang University. In the experiment, 20 OPM-MEG sensors were placed over the bilateral motor cortices on the scalp, and the positions of the sensors were arranged according to the 10–20 system of EEG. A flashing light served as a cue, instructing the subject to perform a movement involving all five fingers of the right hand upon its appearance. The inter-stimulus interval was set to 3 s, and approximately 30 trials were conducted.

The MEG data were band-pass filtered between 3 and 40 Hz, followed by noise removal using homogeneous field correction and independent component analysis (ICA). Then, the continuous data were segmented into epoch data (−1–2.5 s relative to stimulus onset). The epoch data were downsampled to 100 Hz, and MVAR model coefficients were estimated using the CRPF-based method, with the MCF method employed for comparison. The order of the model *p* was set to 4 according to the final prediction error criterion (FPE) [27]. Additionally, the connectivity was measured in the time–frequency domain using the sPDC value, focusing on the frequency range of 3–30 Hz. The sPDC values were averaged across both time and frequency domains to construct effective networks.

#### 2.3.3. Auditory Oddball Experiment

A total of 10 health subjects (8 males and 2 females) participated in this study. The subjects were native Chinese speakers, right-handed, and had no congenital developmental or auditory disorders. All of the subjects provided informed consent and agreed to participate in the experiment. This study was approved by the Research Ethics Committee of Beihang University.

**Figure 2:**
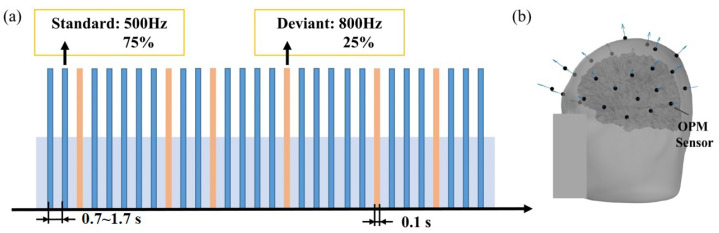
(**a**) Auditory oddball experimental paradigm. (**b**) The position and distribution of OPM magnetometers.

As shown in Figure 2a, the experiment utilized the auditory oddball paradigm, during which the subjects were instructed to fixate on visually interesting images placed in front of them while ignoring the auditory stimulation being presented. A total of 30 OPM magnetometers were symmetrically arranged and fixed to measure the MEG signals. The spatial distribution of the OPM magnetometers covered the temporal, frontal, and parietal lobes. Figure 2b shows the relative positioning of the OPM magnetometers in relation to the subject’s brain. The standard stimulation consisted of a tone at 500 Hz (with an occurrence probability of 75%), while the deviant stimulation was the tone at 800 Hz (with an occurrence probability of 25%). Both standard and deviant stimulations had a duration of 100 ms, with rise/fall times of 5 ms. The inter-stimulus intervals followed a uniform distribution, ranging from 0.7 to 1.7 s, and a total of about 50 deviant stimulations and 200 standard stimulations were presented in the experiment.

The MEG data preprocessing began with band-pass filtering between 2 and 40 Hz. Then, the bad segments were identified and eliminated manually. Furthermore, the homogeneous field correction method was utilized to reduce interference across the entire spectrum. The ICA analysis was implemented to remove artifacts related to heartbeats, blinking, etc.

The continuous MEG data were segmented into epochs. Each trial in each epoch began 0.2 s before the stimulation and ended 0.5 s after the stimulation. Baseline correction was performed using the pre-stimulation data. The auditory event-related magnetic fields were obtained via the superposed average of the standard and deviant stimulation.

A structured-light scanner (SHINING 3D Inc., Hangzhou Zhejiang, China) was applied to record the 3D digitizations of each subject’s helmet. Anatomical magnetic resonance imaging (MRI) was utilized to obtain the brain’s anatomical structure, with a spatial resolution of 1 mm. The three-dimensional image of the helmeted subject was registered with the anatomical structure to determine the position and orientation of the OPM magnetometers relative to the brain. Preprocessing and cortical reconstruction of the subject’s T1-weighted MRI were conducted using Freesurfer [28]. Subsequently, a single-layer boundary element model method was employed to compute the forward solution. In the inverse solution, the dSPM method [29] was then used. In order to facilitate the comparison between the subjects, the cortical surface of each subject was mapped to the “fsaverage” template [30].

The effective brain network was calculated based on the single-trial data. In the calculation, source time series data were extracted from eight brain regions, including the bilateral superior temporal gyrus (STG), bilateral inferior frontal gyrus (IFG), bilateral superior parietal lobule (SPL), and bilateral inferior parietal lobule (IPL), based on the Desikan–Killiany atlas [31] for the construction and analysis of effective brain networks. The CRPF-GC method was used for effective brain network construction. The number of particles was set to 1000, with an initial value of $10^{-4}$, variance $\sigma_{t}^{2}$ set to $2.5\times 10^{-2}$, and the initial cost $C_{t}$ defined as a zero matrix. The order of the model was determined using the FPE criterion [27]. The mean model order for the 10 subjects was 39.5; thus, *p* was set to 40 in the calculations.

Statistical comparisons of the source time series from the STG were conducted at each time point within 0–0.5 s between the standard and deviant stimulation using the Wilcoxon signed-rank test. To analyze the effective brain network of mismatch magnetic fields (MMF), the bootstrapping method [32] was employed to test the differences in the sPDC values between the deviant and standard stimulation.

## 3. Results

### 3.1. Simulation Evaluation

**Figure 3:**
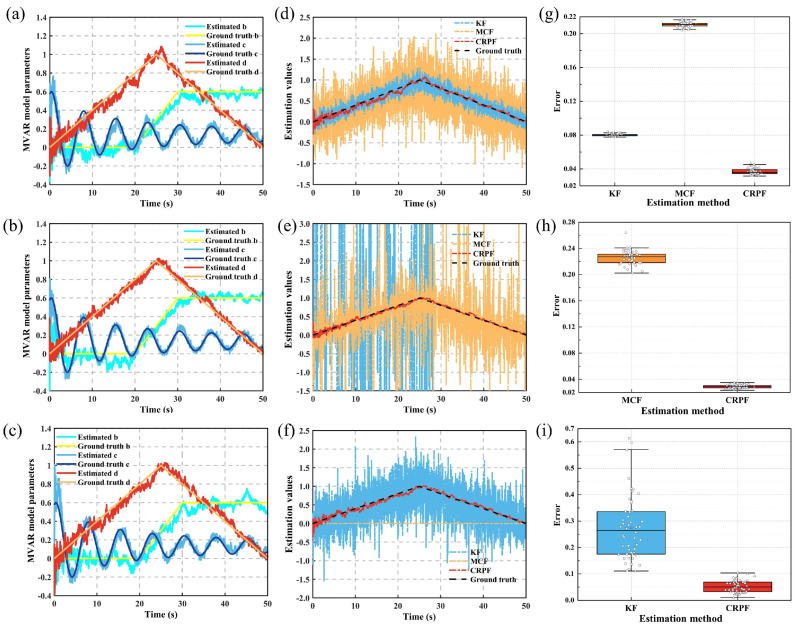
MVAR model coefficient estimation using different noise conditions and filter methods. (**a**–**c**) Comparison between estimated values and ground truth values of MVAR model coefficients using the CRPF method under Gaussian noise, alpha-stable distribution noise, and pink noise, respectively. (**d**–**f**) Estimated values and ground truth values of $d_{t}$ using the KF, MCF, and CRPF methods, respectively. (**g**–**i**) Estimation errors across 50 trials under Gaussian noise, alpha-stable distribution noise, and pink noise, respectively.

The results of the MVAR model coefficient estimation using the KF, MCF, and CRPF methods under Gaussian noise, alpha-stable distribution noise, and pink noise are illustrated in Figure 3. Under all three noise conditions, the estimated values obtained using the CRPF method fluctuated around the ground truth values, demonstrating high estimation accuracy and dynamic tracking capability for the MVAR model coefficients.

Figure 3d–f present a comparison of the estimated values of the MVAR model coefficients $d_{t}$ using the KF, MCF, and CRPF methods. Figure 3g–i show the estimation errors across 50 trials under Gaussian noise, alpha-stable distribution noise, and pink noise, respectively. Table 1 compares the average errors across different noise conditions and filter methods. Under the Gaussian noise condition, the CRPF method exhibited the highest estimation accuracy for the MVAR model coefficients, reducing the average error by 53.4% and 82.4% compared to the KF and MCF methods, respectively. Under the alpha-stable distribution noise condition, the result using the Kalman filter was divergent and failed to track the changes in model coefficients during the later stages of the filter. The average error of the CRPF method was 88.1% lower than that of the MCF method. Additionally, the MCF method produced local outliers when estimating the MVAR model coefficients, which would lead to spurious connectivity values in the connectivity analysis. Under the pink noise condition, the MCF method was also invalid. The average error of CRPF method was 85.8% lower than that of the KF method.

### 3.2. Experimental Analysis

#### 3.2.1. Benchmark Somatosensory Stimulation Analysis

**Figure 4:**
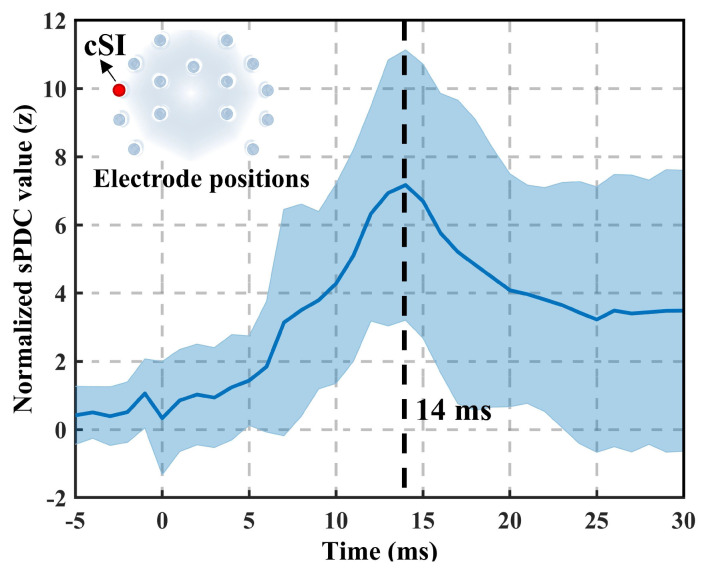
Total outflow sPDC values of the cS1 node during unilateral whisker stimulation in rats.

The cSI receives and processes tactile information transmitted from the skin, muscles, and joints, and it conveys the information to higher-order cerebral centers. In the right whisker somatosensory stimulation experiment, the total outflow sPDC values of the cS1 node (electrode 12) were analyzed in the gamma frequency band. To prevent a single extreme value from dominating the connectivity results, the sPDC values were normalized using z-scores. As shown in Figure 4, the electrode positions are shown in the upper-left figure, and the location of the cS1 node is marked with a red dot. The outflow sPDC values of the cS1 node increased after stimulation, with a peak observed at 14 ms. The results are consistent with previous studies reporting ERP peak latency and connectivity results [25,26].

#### 3.2.2. Finger Movement Analysis

**Figure 5:**
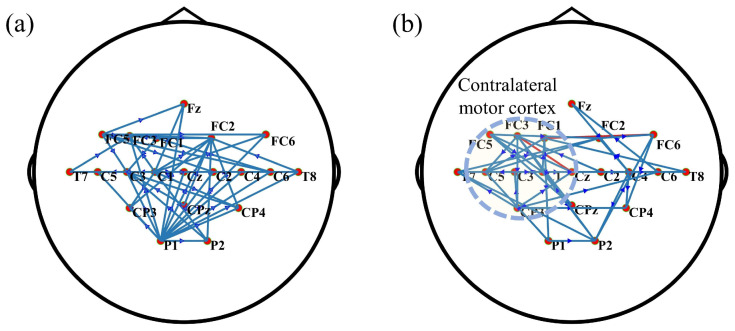
Connections in effective brain networks during finger movement. (**a**) MCF method; (**b**) CRPF method (displays the top 50 connections).

The physiological characteristic of the finger movement experiment is a stronger cortical response in the contralateral motor cortex. The effective brain network of right finger movement using the MCF method is shown in Figure 5a, while the result of the CRPF method is shown in Figure 5b. Notably, the CRPF-GC method revealed dense connectivity between sensors nodes in the contralateral motor cortex, whereas the MCF method failed to exhibit the prominent features. The sensor-level brain network results demonstrate the effectiveness of the CRPF-GC method in analyzing OPM-MEG data.

#### 3.2.3. Auditory Oddball Experiment Analysis

**Figure 6:**
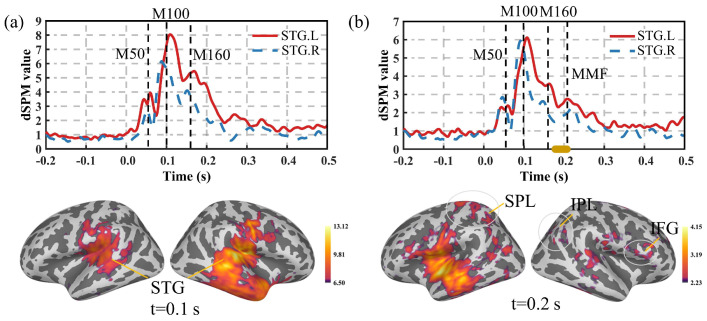
Source activity and time series of bilateral STG. (**a**) Standard stimulation; (**b**) deviant stimulation (significantly different time points are marked with a yellow line along the *x*-axis, p<0.001).

This study utilized OPM-MEG to measure the evoked responses of standard and deviant stimulation in the oddball experiment. The average value of the source activities and the time series of bilateral STG for all of the subjects are shown in Figure 6. The source activity was primarily distributed in the bilateral temporal regions, as well as in the IFG, SPL, and IPL. In the STG, the evoked response to the standard stimulation displayed M50, M100, and M160 components.

MMF is the magnetic counterpart of the mismatch negativity (MMN) in EEG and is typically observed during deviant stimulation in the oddball experiment [33]. Under the deviant stimulation condition, in addition to M50, M100, and M160 components, an MMF component was also detected in the OPM-MEG signals. Statistical analysis indicated a time interval with a significant difference (shown in the yellow line along the *x*-axis in Figure 6b) between the standard and deviant stimulation within the range of 0.179–0.208 s (p<0.001).

**Figure 7:**
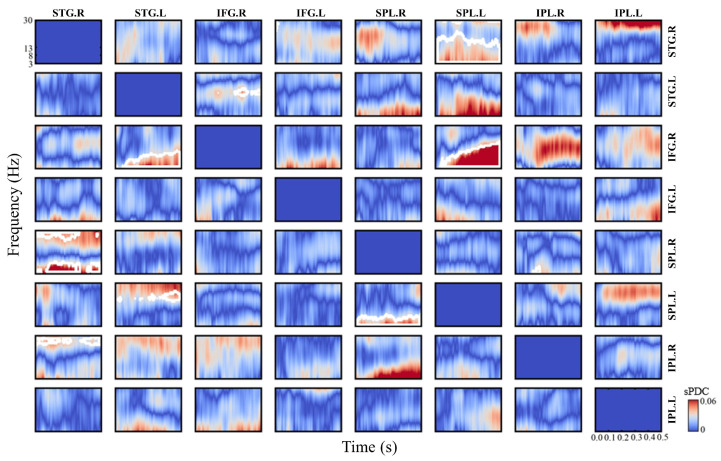
Time–frequency effective brain network of MMF (white boxes represent statistically significant time–frequency points, p<0.05, FDR corrected).

The sPDC value for the MMF was obtained by subtracting the sPDC value of the standard stimulation from that of the deviant stimulation, followed by taking the absolute value, as illustrated in Figure 7 (the white boxed area indicates statistically significant time–frequency points between standard and deviant stimulation, p<0.05, FDR corrected). In Figure 7, the information flows is shown from columns to rows; for example, the time–frequency figure at (row,col) = (2,3) shows information flow at different times and frequencies from the source STG.L to the source IFG.R. Relative to the standard stimulation, the connectivity from the right STG (STG.R) to the right SPL (SPL.R) showed a significant increase, primarily in the theta band (3–8 Hz), in the deviant stimulation. The connectivity from STG.R to the right IPL (IPL.R) exhibited significant increases at time–frequency points located in the beta band (13–30 Hz). The predominant frequency bands for the information flow from the left STG (STG.L) to the right IFG (IFG.R) were identified as theta and alpha bands, while the information flow from STG.L to the left SPL (SPL.L) was primarily in the beta band. Furthermore, the connectivity from SPL.R to SPL.L was significantly increased in the theta and alpha band (8–13 Hz), and the connectivity from SPL.L to STG.R also showed significant increases, mainly in the theta and alpha bands. The information flow from SPL.L to IFG.R included theta, alpha, and beta bands.

**Figure 8:**
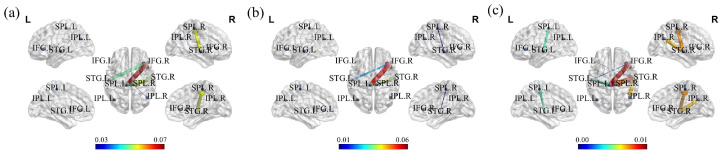
Connections in effective brain networks of MMF with significant differences. (**a**) Connections in the theta band (3–8 Hz); (**b**) Connections in the alpha band (8–13 Hz); (**c**) Connections in the beta band (13–30 Hz).

The time–frequency points with significant differences in Figure 7 were averaged across the temporal dimension to obtain the effective brain network of the mismatch magnetic field in the theta, alpha, and beta frequency bands, as illustrated in Figure 8. Among the connections with significant differences, the STG.L, SPL.L, and IFG.R exhibited strong interhemispheric connectivity across the theta, alpha, and beta bands. Notably, the right IFG served as the primary receiving brain region for information flow in 3–30 Hz.

**Figure 9:**
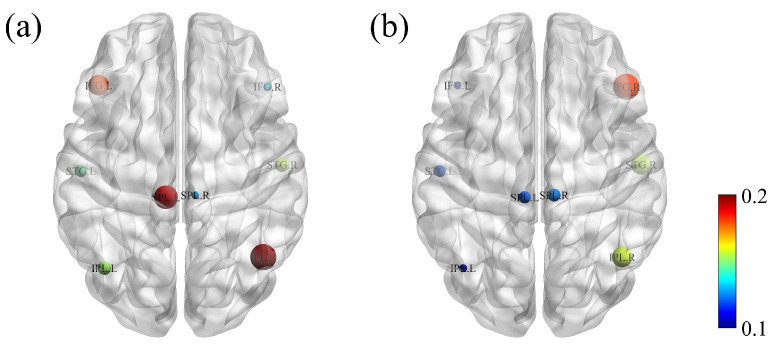
Total inflow sPDC values of MMF. (**a**) MCF method; (**b**) CRPF method.

In the oddball experiment, the total inflow sPDC values of MMF calculated using the CRPF method were further extracted and compared with the results obtained using the MCF method. As shown in Figure 9, the CRPF-GC method clearly identified the IFG.R cortex as exhibiting the highest incoming sPDC values, while the MCF method failed to capture the main brain areas. These findings demonstrate that the CRPF-GC method provides a more comprehensive understanding of brain function.

## 4. Discussion

OPM-MEG is an emerging and developing brain imaging technology [4], and there is currently no established set of technical standards. The feasibility of measuring effective brain networks using OPM-MEG is still under investigation. In the computation of Granger causality, the accuracy of MVAR model coefficient estimation is critical for constructing effective brain networks. This study proposes a CRPF-GC method for estimating MVAR model coefficients under conditions of unknown noise statistical characteristics. The performance of CRPF-GC was evaluated using simulation experiments and benchmark EEG data in rats. This study further used OPM-MEG to measure brain signatures during finger movement and the auditory oddball experiment. The CRPF-GC method was applied to construct the time–frequency effective brain networks. To the best of the authors’ knowledge, this is the first study using OPM-MEG to measure time–frequency effective brain networks.

Electrophysiological signals originating from neural activity in the brain exhibit highly non-stationary characteristics [34]. The causes of non-stationarity in these signals include unstable statistical properties, time-varying spectral components, multiple unknown impulsive non-Gaussian noise, etc. [35,36]. The Kalman filter and its variants have been validated for their effectiveness in constructing effective brain networks from non-stationary neural signals [37], under the assumption that MVAR model noise is Gaussian. However, under real measurement conditions, the neural signals may include time-varying non-Gaussian noise, and the statistical characteristics of the noise are also difficult to determine.

To resolve the issue of poor model coefficient estimation accuracy of MVAR using the Granger causality method for effective networks with unknown noise characteristics, a method for constructing effective brain networks based on CRPF is proposed. In the algorithm, the robustness and accuracy of the filter are enhanced by designing cost functions and risk functions for estimating MVAR model coefficients [16,38]. The simulation results show that the average errors of MVAR model coefficient estimation using the CRPF method are reduced by 53.4% and 82.4% compared to the KF and MCF methods under Gaussian noise conditions, respectively. The average errors are reduced by 88.1% and 85.8% compared to the MCF method under non-Gaussian alpha-stable distribution noise and the KF method under pink noise conditions, respectively. These results indicate that the proposed algorithm improves the accuracy of MVAR model coefficient estimation.

In the benchmark somatosensory stimulation data in rats, the peak of the total outflow sPDC value at cS1 coincided with the ERP peak latency and the connectivity peak time reported in previous studies [25,26]. This demonstrates the capability of the CRPF method to characterize the time–frequency features of effective brain networks. In the finger movement experiment measured using OPM-MEG, compared to the MCF method, the CRPF method revealed effective brain networks that captured the lateralized response of the contralateral hemisphere, aligning more closely with known physiological patterns. These findings suggest that the CRPF method can reconstruct dynamic effective networks and critical connectivity patterns with higher temporal–frequency resolution. This capability enables CRPF to provide novel insights into the rapid dynamics of neural interactions in the brain.

The oddball paradigm is a widely used experiment in cognitive neuroscience that typically includes high-probability standard stimulation and low-probability deviant stimulation. It investigates the brain’s response to infrequent and novel deviant stimulation. When occasional deviant stimulations are presented within a series of repetitive standard stimulations, the MMF is elicited, even when subjects are instructed to ignore the stimulation and perform no specific task. MMF reflects the automatic processing and recognition of stimulation characteristics in the brain [33]. Therefore, this holds important diagnostic value for patients who are unconscious or exhibit poor compliance [39]. MMN usually occurs in the latency range of 0.15–0.25 s [33,40]. In this study, the time intervals with significant differences between standard and deviant stimulation inthe OPM-MEG measurements were within the range of 0.179–0.208 s, which aligns with previous research. These results demonstrate the effectiveness of OPM-MEG in capturing subtle event-related neural signals.

The brain network detection of MMF relies on theta, alpha, and beta band effective connectivity [41]. In this study, the time–frequency effective brain network related to the MMF clearly indicates critical time–frequency windows and information flow patterns of inter-regional brain interactions within the theta, alpha, and beta bands. In disorders such as schizophrenia, elevated connectivity in the frontal lobe is observed [42]. The inferior frontal gyrus is also identified as the primary brain region responsible for generating the MMF [43]. The effective brain network constructed using the CRPF-GC algorithm demonstrates that the right inferior frontal gyrus is the major receiving region for information flow, consistent with previous effective brain network analyses using dynamic causal modeling method [44,45]. The observed increase in connectivity between the hemispheres (e.g., SPL.R → IFG.R) suggests the involvement of interhemispheric integration in the MMF process. In this study, differences in pitch frequency between standard and deviant stimulation led to a bias in the effective connectivity network toward the right hemisphere, aligning with fMRI source activation results [43]. These findings reveal the hemispheric differences in processing auditory feature information. Overall, the results validate the feasibility of constructing effective brain networks using OPM-MEG.

OPM-MEG is an emerging technique for measuring neural magnetic signals. Demonstrating its ability to quantify effective brain networks lays the foundation for its broad application. As a “zero-field” magnetometer, OPM-MEG is inherently more susceptible to noise interference [46]. The CRPF method provides a robust framework for constructing time–frequency MVAR models in non-stationary data, even without prior knowledge of noise characteristics. It enables precise characterization of dynamic changes at specific frequencies, network configuration features, and event-related states on a millisecond timescale. This method offers a valuable tool for exploring the neural mechanisms underlying sensory, motor, and cognitive tasks while mitigating noise impacts in the construction of effective brain networks using OPM-MEG.

CRPF is a variant of particle filters, where the number of particles and the resampling strategy play a critical role in determining computational accuracy. Developing adaptive strategies for selecting particle numbers and resampling methods based on the scale of MVAR model coefficients represents a critical aspect for future investigation. Furthermore, more accurate effective network construction could support refined characterization of neural research and clinical disease. For instance, it may facilitate the classification of clinical characteristics in patients with neurological disorders [47], staging of progressive neurodegenerative diseases [48], or applications in motor control for brain–computer interfaces [49].

## 5. Conclusions

This study proposes the CRPF-GC method for constructing effective brain networks. In simulations, the average estimation errors of the MVAR model coefficients using the CRPF were reduced by 53.4% and 82.4% compared to the KF and MCF methods under Gaussian noise conditions, respectively. In the presence of non-Gaussian alpha-stable distributed noise, the average error of the CRPF was 88.1% lower than that of the MCF method. Under the pink noise condition, the MCF method was also invalid. The average error of the CRPF method was 85.8% lower than that of MCF method. These results indicate that the CRPF-GC algorithm improves the accuracy of MVAR model coefficient estimation. In the rat somatosensory stimulation benchmark data and human finger movement experiments, the results obtained using CRPF-GC were consistent with previous studies and physiological characteristics. In the oddball experiment, the MMF component during deviant stimulation was detected in the OPM-MEG data. The effective brain networks of MMF reveal that the right inferior frontal gyrus is the primary brain region for information inflow. In summary, the results of the simulation and measurements validate the effectiveness of the CRPF-GC method and demonstrate the feasibility of OPM-MEG in measuring time–frequency effective networks. The study also provides valuable insights for the mechanistic understanding of effective networks in the brain.

## Figures and Tables

**Table 1:** Comparison of average errors under different noise conditions and filter methods (mean value ± standard deviation).

Noise Conditions	Estimation Method		
	KF	MCF	CRPF
Gaussian noise	0.0801 ± 0.0013	0.2106 ± 0.0027	0.0371 ± 0.0034
Alpha-stable distribution noise	–	0.2260 ± 0.0106	0.0269 ± 0.0081
Pink noise	0.2416 ± 0.1405	–	0.0344 ± 0.0104

## Data Availability

The data, aside from the data published in this manuscript, are not publicly available due to privacy restrictions.

## Supplementary Materials

The following supporting information can be downloaded at: https://www.mdpi.com/article/10.3390/bioengineering11121258/s1, Table S1: Error and calculation time vary with the number of particles.
