# Supplementary material for: Cost-Reference Particle Filter-Based Method for Constructing Effective Brain Networks: Application in Optically Pumped Magnetometer Magnetoencephalography
Source: Bioengineering (Basel). 2024 Dec 12;11(12):1258. doi: 10.3390/bioengineering11121258 (PMC11673604; doi:10.3390/bioengineering11121258)
Supplement: Supplementary file 1 [file bioengineering-11-01258-s001.zip › bioengineering-3324392-supplementary.pdf]

Supplementary Materials

- **The influence of particle number on cost-reference particle filter**

The number of particles represents a trade-off between computational accuracy and computation time. An increase in the number of particles directly leads to an increase in computation time, because each particle requires state updates, weight calculations, and resampling operations. Using Gaussian noise as an example, we have analyzed how the estimation error changes with the increase in the number of particles, as shown in the Table 1. below. It can be observed that after increasing to 1000 particles, the trend of decreasing average estimation error diminishes with the addition of more particles, such as using 1500 particles. In this study, we selected the number of particles to be 1000. In future calculations, if conditions permit, more particle numbers can be used.

Table S1. Error and calculation time vary with the number of particles

Particles number	100	500	750	1000	1500
Error	0.0521	0.0451	0.0460	0.0368	0.0356
Time(s)	12.95	54.18	79.82	100.8	145.25
